# Case report: A rare case of coexisting Waldenstrom Macroglobulinemia and B-cell acute lymphoblastic leukemia with KMT2D and MECOM mutations

**DOI:** 10.3389/fimmu.2022.1001482

**Published:** 2022-10-17

**Authors:** Lingling Wang, Jiao Tang, Jun Feng, Yongfen Huang, Yuexin Cheng, Hao Xu, Yuqing Miao

**Affiliations:** ^1^ Department of Hematology, The First People’s Hospital of Yancheng, The Yancheng Clinical College of Xuzhou Medical University, Yancheng, China; ^2^ Department of Neurology, The First People’s Hospital of Yancheng, The Yancheng Clinical College of Xuzhou Medical University, Yancheng, China

**Keywords:** Waldenstrom Macroglobulinemia, acute lymphoblastic leukemia, B cell, plasma cells, IgM, MYD88, KMT2D, MECOM

## Abstract

**Background:**

Waldenstrom Macroglobulinemia (WM) is a rare and indolent lymphoma of B-cell origin characterized by elevated monoclonal IgM, with MYD88L265P mutation and CXCR4 mutation as common molecular alterations. B-cell Acute Lymphoblastic Leukemia (B-ALL) is clinically heterogeneous, characterized by abnormal proliferation and aggregation of immature lymphocytes in the bone marrow and lymphoid tissue. WM and ALL are hematologic malignancies of B-cell origin with completely different clinical manifestations and biological features. KMT2D and MECOM mutations are very rare in ALL and usually indicate poor disease prognosis. The coexistence of WM and ALL with KMT2D and MECOM mutations have not been reported.

**Case presentation:**

A 74-year-old female patient was diagnosed with WM in July 2018 and received four cycles of chemotherapy of bortezomib and dexamethasone. In November 2018, she received immunomodulator thalidomide as maintenance therapy. In November 2020, Bruton’s Tyrosine Kinase inhibitors (BTKi) has been introduced into the Chinese market and she took zanubrutinib orally at a dose of 80 mg per day. The disease remained in remission. In December 2021, she presented with multiple enlarged lymph nodes throughout the body. Bone marrow and next-generation sequencing (NGS) suggested the coexistence of WM and B-ALL with KMT2D and MECOM mutations. The patient was treated with zanubrutinib in combination with vincristine and dexamethasone, after which she developed severe myelosuppression and septicemia. The patient finally got remission. Due to the patient’s age and poor status, she refused intravenous chemotherapy and is currently treated with zanubrutinib.

**Conclusions:**

The coexistence of WM and B-ALL is very rare and has not been reported. The presence of both KMT2D and MECOM mutations predicts a poor prognosis and the possibility of insensitivity to conventional treatment options. BTKi achieves its anti-tumor effects by inhibiting BTK activation and blocking a series of malignant transformations in B-cell tumors. In addition, it also acts on T-cell immunity and tumor microenvironment. Combination therapy based on BTKi may improve the prognosis of this patient.

## Background

Under repeated stimulation by antigens, B-lymphocytes undergo proliferation and differentiation and are transformed into plasma cells. WM is a rare disease of plasma cell origin, characterized by the accumulation of malignant lymphoplasmacytes in the bone marrow and other organs, and the secretion of monoclonal IgM (1, 2). WM is mostly seen in the elderly and is an indolent tumor with family heritability. The disease is incurable, with significant heterogeneity and a variable prognosis, and some patients may progress to other types of hematologic malignancies by the advanced stages of the disease. Once disease transformation occurs, the prognosis is very poor. WM and B-ALL share a common progenitor cell origin. They are two completely independent hematologic malignancies. The clinical manifestations of ALL include signs of normal hematopoietic failure of the bone marrow caused by infiltration of bone marrow tissue with leukemic cells (e.g., anemia, infection, hemorrhage, etc.) and abnormalities caused by extramedullary infiltration of leukemic cells (e.g., lymph nodes, hepatosplenomegaly, etc.) (3). Herein, we report the first case of coexisting WM and B-ALL. This patient had a 3-year history of WM and revisited our hospital for generalized lymph node enlargement. Bone marrow examination and NGS suggested the coexistence of WM and B-ALL with KMT2D and MECOM mutations.

## Case presentation

**Figure 1:**
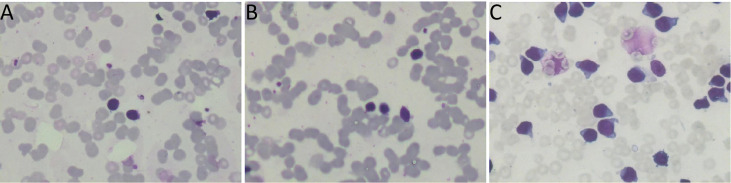
**(A, B)** the bone marrow smear suggested 78% lymphoplasmacytic lymphoma cells. **(C)** The bone marrow smear was significantly active in proliferation. Primitive cells accounted for 94.5% of the total number of cells.

In July 2018, a 74-year-old female patient was admitted to the Department of Hematology in our hospital and complained of weakness. The patient had a history of chronic nephritis. Physical examination did not reveal enlarged lymph nodes, splenomegaly, or hepatomegaly. The hemoglobin level was decreased at 86g/L, leukocyte 2.0×10^9^/L, and platelets 64×10^9^/L. Biochemical showed globulin at 26.4g/L, albumin at 31.2g/L, lactate dehydrogenase (LDH) at 99.3U/L, β2-microglobulin at 4.34mg/L, and an elevated monoclonal IgM at 9.86g/L. The peripheral blood smear suggested 59% lymphocytes. The bone marrow smear suggested 78% lymphoplasmacytic cells (**Figures 1A, B**). The MYD88L265P mutation was positive. A serum protein electrophoresis (SPEP) and immunofixation electrophoresis (IFE) showed a monoclonal IgM with λ light chain restriction. Bone marrow immunophenotype suggested that lymphoplasmacytic cells occupied about 43.8% of nuclear cells, of which CD19 positive cells occupied about 6.5% of nuclear cells, expressing HLA-DR, CD19, CD20, CD22, CD25, CD200, cIgM, and Lambda. Karyotypic showed 46, XX, del(20)(q11) [5]. Combined with the clinical manifestations and related tests, the diagnosis of WM was made. Four cycles of chemotherapy with bortezomib and dexamethasone were administered, followed by oral thalidomide maintenance therapy. Blood tests during follow-up showed a significant decrease in white blood cells, and the patient’s bone marrow was retested several times, suggesting significant hypoproliferation. In November 2020, the patient chose zanubrutinib as maintenance therapy. Based on the premise that the patient had low myeloproliferation, zanubrutinib was adjusted to 80 mg daily, considering that standard doses of zanubrutinib may aggravate myelosuppression.

In December 2021, the patient was transferred to the local hospital because of worsening weakness. Bone marrow smear indicated abnormal cells accounted for 37.2%. The immunophenotype was as follows. The monoclonal plasma cells accounted for about 25.6% of nuclear cells, and another 6.3% of the mature monoclonal B lymphocyte was seen, expressing cKAPPA, CD81, CD19, CD138, CD38, CD45, and negative for cLAMBDA, CD56, CD117, CD27. The gene was positive for MYD88 mutation and negative for CXCR4. FISH suggested IgH/CCND1 was negative. No abnormalities of 13q-, 17p-, +1q21, t(4;14)(14;16), or t(14;20) were detected. Positron Emission Tomography-Computed Tomography (PET-CT) suggested multiple enlarged lymph nodes of variable size in both inguinal regions, bilateral cervical, mediastinal, hilar, parietal, and retroperitoneal. The IgH rearrangement was positive. But the patient did not receive further diagnosis or treatment.

**Figure 2:**
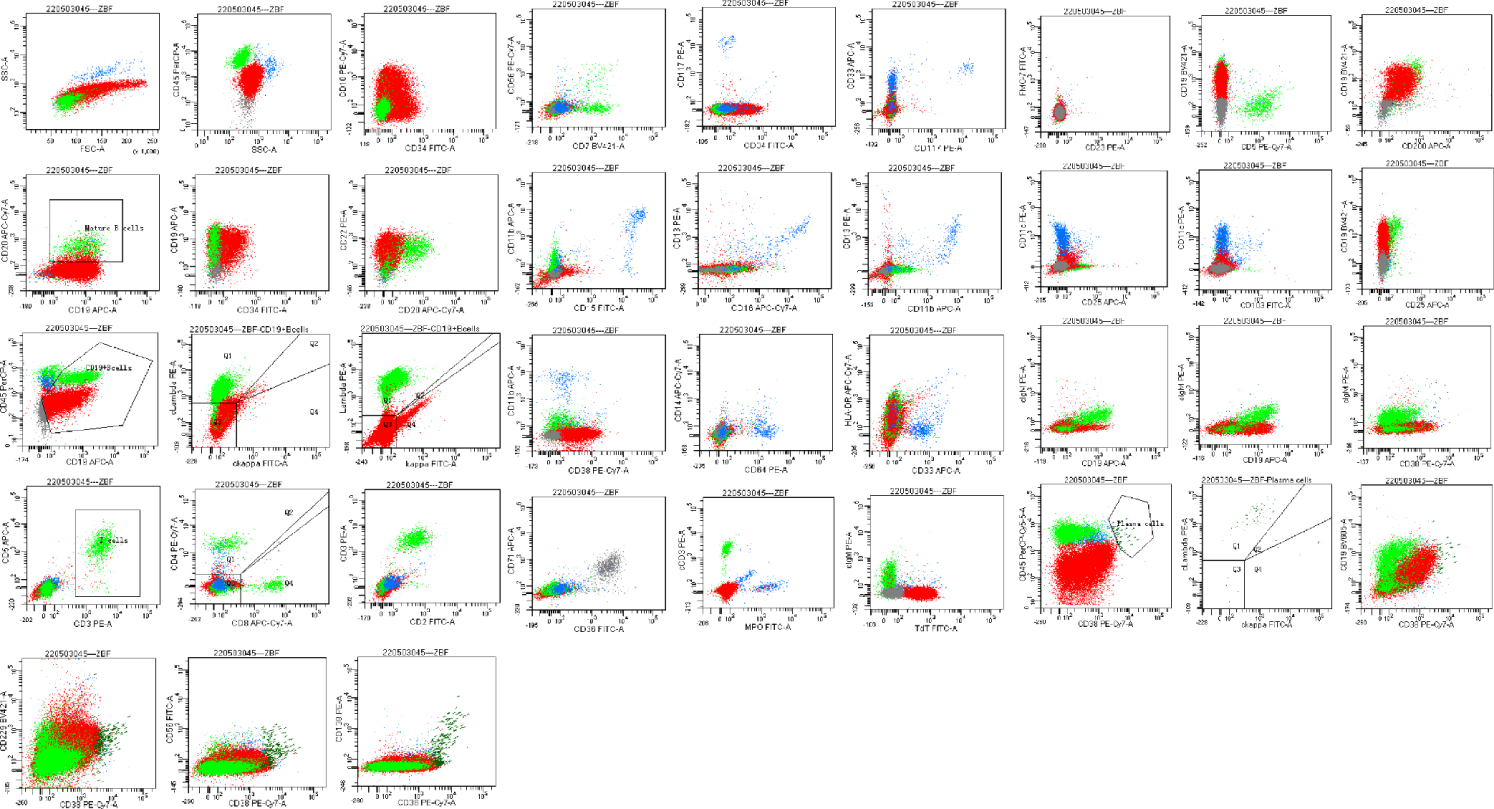
Bone marrow flow cytometry revealed an abnormal cell population occupying about 82.1% of nuclear cells, expressing CD19, CD22, and TdT. Some cells expressed HLA-DR, CD10, CD34, and CD38, not expressed cIgM, indicating abnormal naive B lymphocytes. Cells with CD45+CD19+ occupying about 8.0% of nuclear cells, expressing HLA-DR, CD19, CD20, CD22, sIgM, cIgM, cLambda, Lambda, suggested aberrant monoclonal B lymphocytes. Cells with CD45dimCD38bright occupied about 0.1% of nuclear cells, expressing CD19, CD38, CD56, CD138, CD229, cLambda, suggested aberrant monoclonal plasma cells.

**Figure 3:**
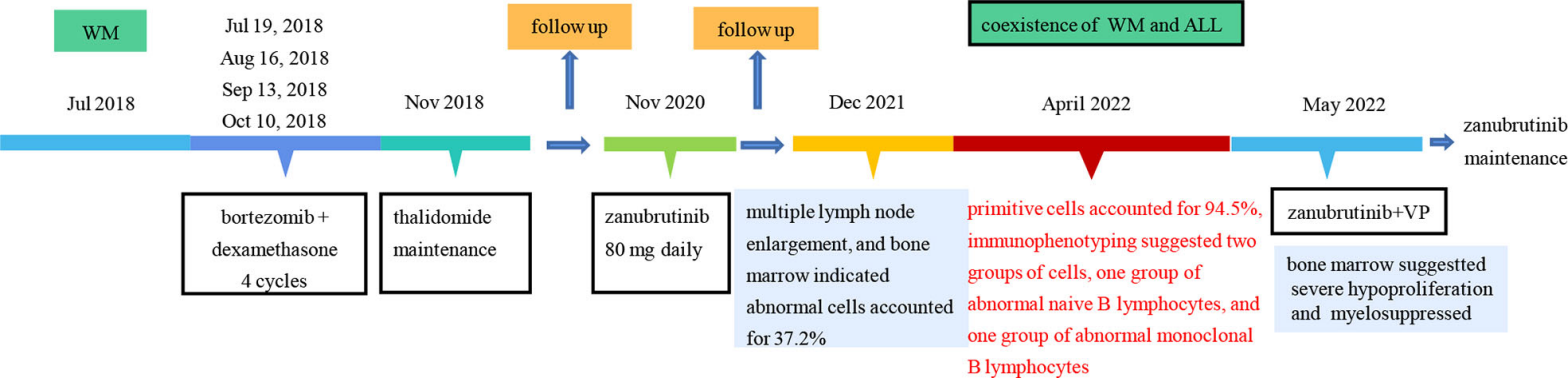
The treatment timeline of the patient.

In April 2022, the patient was transferred to our hospital for further treatment. Physical examination revealed splenomegaly. The white blood cell count was 22.09×10^9^/L, hemoglobin 81g/L, and platelets 36×10^9^/L. Biochemistry showed total protein 54.4g/L, albumin 33.9g/L, globulin 20.5g/L, LDH 266.5U/L, IgG 7.41g/L, and IgM 0.71g/L. The peripheral blood smear suggested lymphocytes account for 94%. CT showed multiple enlarged lymph nodes in the neck, mediastinum, and retroperitoneum. The SPEP and IFE showed a monoclonal IgM with λ light chain restriction. Bence-Jones protein was negative. The bone marrow smear was significantly active in proliferation. Primitive cells accounted for 94.5% of the total number of cells, and their morphology was characterized by cells of unequal size, which were round-like or oval. The cytoplasm was medium in volume, sky blue with darker margins and no granules. The nucleus was irregular, with depression, folding, and kidney shape. The nucleoplasm was finely granular and evenly distributed. The POX staining was negative (**Figure 1C**). Bone marrow flow cytometry revealed an abnormal cell population occupying about 82.1% of nuclear cells, expressing CD19, CD22, and TdT. Some cells expressed HLA-DR, CD10, CD34, and CD38, not expressed cIgM, indicating abnormal naive B lymphocytes. Cells with CD45+CD19+ occupying about 8.0% of nuclear cells, expressing HLA-DR, CD19, CD20, CD22, sIgM, cIgM, cLambda, Lambda, suggested aberrant monoclonal B lymphocytes. Cells with CD45dimCD38bright occupied about 0.1% of nuclear cells, expressing CD19, CD38, CD56, CD138, CD229, and cLambda, suggesting aberrant monoclonal plasma cells (**Figure 2**). NGS suggested missense mutation in MYD88L265P and nonsense mutation in KMT2D. MECOM mutation was also detected. The IKZF, TP53, KMT2A were all negative. Myeloid leukemia-related mutations such as FLT3, NRAS, KRAS, and DNMT3A were negative. Mutations associated with T-ALL such as NOTCH1 and CDKN1/2 were also negative. Karyotypic analysis showed 45, XX, add (4)(q31), -9[15]. The patient was diagnosed with the coexistence of WM and B-ALL with KMT2D and MECOM mutations. The patient was of advanced age, with poor physical status and heavy anemia with extreme thrombocytopenia. The choice of the treatment regimen was fully balanced between WM and B-ALL, and we finally proposed a treatment plan of zanubrutinib in combination with VP (vincristine plus dexamethasone). One month later, the patient’s bone marrow was retested, suggesting severe hypoproliferation and myelosuppressed. Measurable residual disease (MRD) suggested two groups of abnormal cells, a group of CD45+CD19+ cells, which occupied 23.37% of the nucleated cells, expressing CD19, CD20, Lambda, cLambda, considered as abnormal monoclonal B lymphocytes. The other group was CD45dimCD19+ cells, occupying 0.24% of nuclear cells, expressing CD10st, CD20, CD34, CD38, and CD58, considered abnormal naive B lymphocytes. During this period, the patient had a recurrent high fever and was considered to have a secondary infection after chemotherapy. Blood cultures and NGS tests did not detect positive findings. Carbapenems, vancomycin, and voriconazole were used, and the temperature peaks finally decreased significantly. The patient’s physical status was very poor which made it difficult to undergo the next phase of chemotherapy. Therefore, only zanubrutinib was administered as maintenance therapy (**Figure 3**). The patient was last hospitalized in May 2022 and had been followed up for 3 months since discharge. The patient received regular blood tests. The patient’s blood count in Aug 2022 indicated the white blood cell of 2.49×10^9^/L, platelets of 66×10^9^/L, and hemoglobin of 96g/L. Despite the patient’s poor condition, there were no significant signs of infection or bleeding. Since the patient refused intravenous chemotherapy, the current treatment is based on oral BTK inhibitors.

## Discussion and conclusions

The diagnosis of WM is based on the following aspects. First, the clinical manifestations of the patient. Since WM is a B-cell-derived NHL characterized by lymphoplasmacytic invasion of the bone marrow and secretion of monoclonal IgM, the patient will present with lymphoma-like systemic clinical symptoms, as well as high IgM levels leading to hyperviscosity syndrome and cryoglobulinemia. Second, in addition to clinical manifestations, monoclonal IgM must be detected in the serum, and lymphoplasmacytic cells should be seen in the bone marrow. The typical immunophenotype are CD22 (+), CD25 (+), CD103 (-), CD3 (-), etc. MYD88L265P mutation is an important but non-specific marker for the diagnosis and differential diagnosis of WM, with a 90% incidence, and CXCR4 mutation is also present in 30% of patients (4). In addition, the diagnosis of WM needs to be differentiated from other B-cell lymphomas (5). WM is a rare and indolent disease with slow disease progression, and therefore this group of patients tends to have longer survival. Indications for treatment include B symptoms, i.e., fever and night sweats, weight loss, etc., symptomatic hyperviscosity (patients with retinal hemorrhage, CNS symptoms), peripheral neuropathy, organomegaly, amyloidosis, cold agglutinin disease, cryoglobulinemia, disease-related anemia, extramedullary lesions, CNS lesions (Bing-Neel syndrome), giant lymph nodes, or when there is evidence of disease transformation (6).

The incidence of ALL is “bimodal”, with the highest incidence in children, followed by a gradual decrease, and then a gradual increase after the age of 60. With advances in treatment, the prognosis for this type of leukemia is better in pediatric patients, with a 5-year survival of up to 90% in children aged 1-10 years old (7). In contrast, the 5-year survival of patients >60 is only about 10%, with a poorer prognosis, which is also significantly associated with genetic abnormalities in patients. The main genetic abnormalities in pediatric patients are indicators of good prognosis such as hyperdiploidy and t (12,21). With increasing age, these abnormalities gradually decrease, while indicators of poorer prognosis such as t (9,22) gradually increase. There are still 10% to 20% unknown genetic abnormalities in patients of different ages that need to be further elucidated. The development of B-ALL is a multistep pathogenetic process. Genetic variations and polymorphisms in lymphoid progenitor cells increase the susceptibility to developing leukemia. Initiation of leukemic differentiation includes the presence of chromosomal aneuploidy, genetic recombination due to chromosomal translocations, and gene mutations. In concert with other abnormal genes such as oncogenes, kinase, and ras signaling pathway genes, alterations in transcript factors, and epigenetically related genes, leukemia occurs in cellular tissues during the early B-cell production phase. Relapse after disease treatment may be associated with the proliferation of pre-existing subclones and the development of drug-resistant genes.

Cases combining two malignancies of the hematological system are very rare, and to our knowledge, there are no literature reports of the coexistence of WM and B-ALL. Herein, we report the first rare case of coexistence of WM and B-ALL, analyze the underlying pathogenesis, and propose our therapeutic regimen. This was a 74-year-old female patient who was diagnosed with WM in July 2018, treated with bortezomib in combination with dexamethasone for 4 cycles, achieved remission, and then received thalidomide maintenance therapy. After the BTK inhibitor was introduced into the Chinese market, she received maintenance treatment with zanubrutinib. In April 2022, a CT examination suggested multiple enlarged lymph nodes throughout the body. Bone marrow examination suggested the coexistence of WM and B-ALL. We believe that this patient’s diagnosis of coexistence of WM and B-ALL rather than WM secondary to B-ALL is based on the following basis. First, the bone marrow immunophenotyping of this patient suggested two groups of cells. One group of abnormal naive B lymphocytes, accounting for 82.1%, with an immunophenotype consistent with B-ALL, and another group of abnormal monoclonal B lymphocytes, accounting for 8.0%. In addition, a bone marrow smear showed 94.5% of primitive cells with POX staining negative. Therefore, the diagnosis of ALL was clear. Second, the NGS examination suggested the MYD88L265P mutation was positive. The SPEP and IFE suggested monoclonal IgM with λ light chain restriction. This suggested that the diagnosis of WM was still valid. Third, no literature reports and retrospective data analysis suggested that bortezomib and thalidomide could induce a second tumor within a short time. Reports of BTK inhibitors inducing a second tumor are even rarer. In addition, this patient was treated orally with a reduced dose of zanubrutinib, which has a much lower cumulative toxic effect. Therefore, we believe that the development of B-ALL in this patient was not related to the history of prior drug use.

We speculate that the pathogenesis of WM and B-ALL appearing simultaneously in this patient were as follows. First, there is a common etiology of leukemia and lymphoma, and retroviruses have been shown in recent years to be oncogenic viruses that induce leukemia, lymphoma, and mammary tumors in reptiles and primate mammals. Second, WM and B-ALL share a common progenitor cell origin. Thirdly, chromosomal abnormalities and the presence of oncogenes allow malignant cloning of pluripotent stem cells with potential pluripotent differentiation, with different sites and manifestations of lesions at different times. Chromosomal damage can activate oncogenes to induce replication and produce abnormal clones that contribute to tumorigenesis. Fourthly, WM patients all have different degrees of immune deficiency, The immune system could be suppressed after chemotherapy, which makes the immune surveillance of tumor cells out of control, thus providing conditions for the proliferation of tumor cells and leading to the occurrence of other malignant tumors in the hematological system. Fifthly, clonal evolution may also be one of the pathogenesis. Clonal heterogeneity is progressively complicated by the development of disease, clonal screening for therapy, and changes in the tumor microenvironment. This eventually leads to disease progression. The Second Primary Tumor(SPT) is the occurrence of two or more separate primary tumors in the same organ, in paired organ tissues, in different parts of the same system, and in organ tissues of different systems, either simultaneously or sequentially. The pathogenesis of SPT is still unclear. With the progress of medical development, people have more opportunities to seek medical treatment actively, and the implementation of a multidisciplinary diagnosis and treatment model, which makes the number of tumor survivors increase, thus leading to the increase in second tumor incidence. The persistence of exogenous carcinogenic factors, genetic susceptibility, the presence of immune deficiency, and pre-treatment factors are all possible pathogenic mechanisms.

WM is an indolent lymphoma and ALL is an aggressive tumor. It is very rare for a patient to have WM combined with ALL at the same time. Since both diseases are of B-cell origin, we finally chose a treatment regimen of BTKi combined with VP after a comprehensive assessment of the patient’s physical status and age. In addition to directly inhibiting B cells, BTKi also normalizes the number of T cell subsets, regulates T cell differentiation and function, and promotes T cell differentiation in an immune-friendly direction, thereby enhancing T cell-mediated immune responses, anti-tumor activity, and immune surveillance (8, 9). BTKi enhances the immune effect of Th1 and promotes the secretion of IL-2, IFN-γ, and TNF, which enhances the toxic effect on tumor cells. It also down-regulates PD-1 expression on CD8+/CD4+ T cells, decreasing the suppressive effect on T cells and enhancing immunity. In addition, it can reduce a variety of biomolecules that are detrimental to anti-tumor immunity, such as IL-10 and IL-6, and alleviate the immunosuppressive effects of these molecules. Through the improvement of T cell differentiation and function, BTKi can synergize multiple pathways comprehensively to regulate the tumor microenvironment, further enhance immune function and inhibit tumor growth and proliferation (10).

The treatment goal for this patient is to pursue rapid symptom relief, reduce the risk of organ damage and tumor load, and prolong the patient’s survival. Mitigating treatment-related toxicity while avoiding late-stage complications. The selection of regimens for this patient should be individualized, based on the patient’s factors, related symptoms, genomic characteristics, and the efficacy and safety of the regimen. This patient had difficulty tolerating intense chemotherapy. The prognosis for WM combined with ALL is very poor and has not been reported in the literature. There is no uniform treatment protocol. Our treatment aims to relieve the patient’s symptoms, improve quality of life, and reduce drug-related toxicities and side effects. Therefore, BTK inhibitor combined with VP regimen chemotherapy was chosen at that time.

NGS of this patient revealed KMT2D and MECOM mutations. KMT2D encodes histone H3K4 methyltransferase and is one of the most commonly mutated genes in tumor patients (11). Further analysis revealed that KMT2D has an important role in transcriptional regulation and DNA damage repair. Studies showed that KMT2D mutation leads to a significant decrease in H3K4 monomethylation levels in tumor cells, and collisions between transcriptional and replication forks in KMT2D mutation cells result in increased levels of genomic DNA damage and mutational load, as well as elevated transcriptional instability (12). Dr. Wang et al. systematically investigated the relationship between 56 gene mutations and immune checkpoint treatment response using a CRISPR-GEMM screening model. Through screening and validation, tumors with KMT2D mutation were found to be more sensitive to PD-1 therapy (13). MECOM genes primarily control embryonic development and can be aberrantly expressed or generate fusion genes by 3q rearrangements, MLL translocations, chromosome 7, or interactions with other genes. The MECOM gene is aberrantly expressed in some ALL, AML, and CML, and is associated with poor prognosis (14). The leukemogenic mechanisms include epigenetic modifications, regulation of transcript, modulation of signaling pathways, and enhancement of leukemic cell adhesion, proliferation, colony formation, and resistance to apoptosis. The presence of both KMT2D and MECOM mutations in this patient predicted a poor prognosis and the possibility of insensitivity to conventional treatment.

The coexistence of WM and B-ALL is very rare. BTK is a key factor in the proliferation of B-cell tumors. BTKi achieves its anti-tumor effects by inhibiting BTK activation and blocking a series of malignant transformations in B-cell tumors. In addition, it also acts on T-cell immunity and tumor microenvironment. Combination therapy based on BTKi may improve the prognosis of this patient. This is the first reported case of WM combined with ALL, which is very rare. The treatment for this patient was determined by combining multiple factors such as the patient’s physical status, age, and drug accessibility. The effectiveness of this treatment regimen needs to be followed up.

## Data availability statement

The original contributions presented in the study are included in the article/supplementary material. Further inquiries can be directed to the corresponding author.

## Ethics statement

The studies involving human participants were reviewed and approved by The First people’s Hospital of Yancheng. The patients/participants provided their written informed consent to participate in this study. Written informed consent was obtained from the individual(s) for the publication of any potentially identifiable images or data included in this article.

## Author contributions

LW and JT participated in manuscript writing and data analysis. JF and YH collected the clinical data. YC, HX, and YM participated in specialized guidance and clinical treatment. All authors contributed to the article and approved the submitted version.

## Funding

This work was supported by Yancheng Municipal Health Commission (Grant Number YK2020004).

## Conflict of interest

The authors declare that the research was conducted in the absence of any commercial or financial relationships that could be construed as a potential conflict of interest.

## Publisher’s note

All claims expressed in this article are solely those of the authors and do not necessarily represent those of their affiliated organizations, or those of the publisher, the editors and the reviewers. Any product that may be evaluated in this article, or claim that may be made by its manufacturer, is not guaranteed or endorsed by the publisher.
